# Circulating miR-326 could serve as a predictive biomarker for response to neoadjuvant chemotherapy in locally advanced cervical cancer

**DOI:** 10.3389/fonc.2022.1036710

**Published:** 2022-11-09

**Authors:** Kangni Zou, E. Yang, Tao Cui, Zhengyu Li

**Affiliations:** ^1^ Department of Gynecology and Obstetrics, West China Second University Hospital, Sichuan University, Chengdu, China; ^2^ Key Laboratory of Birth Defects and Related Diseases of Women and Children, Sichuan University, Ministry of Education, Chengdu, China

**Keywords:** microRNAs, locally advanced cervical cancer, neoadjuvant chemotherapy, predictive biomarker, molecular

## Abstract

**Background:**

Clinically, few patients with locally advanced cervical cancer (LACC) are insensitive to neoadjuvant chemotherapy (NACT). Recent studies have reported that circulating microRNAs (miRNAs) may be involved in the response to NACT. The aim of this study was to discover the potential miRNAs that can predict the response to NACT in LACC.

**Methods:**

Pair-matched blood samples of 39 LACC patients before and after receiving NACT were collected. Seven paired samples were used for microRNA microarray analysis. Targeted miRNAs were selected by bioinformatics analysis and were validated by quantitative reverse transcription–polymerase chain reaction (qRT-PCR). All 39 patients were assigned into either the responders group or the non-responders group after NACT. The predictive performance of selected microRNA was evaluated by sensitivity, specificity, accuracy, and the area under the receiver operating characteristic (ROC) curve.

**Results:**

A total of 17 miRNAs downregulated before NACT and upregulated after NACT were selected according to microarray analysis in our previous study, and miR-326 and miR-376a-3p were selected for further exploration. According to the responses and the evaluation criteria, 25 patients reached partial response (PR) and 14 patients remained stable. Further qRT-PCR analysis showed that miR-326 significantly downregulated before NACT and upregulated after NACT in 12 responders (*p* = 0.02). The expression of miR-376a-3p showed no statistical difference before and after NACT in these 12 responders. Then, miR-326 provided an AUC-ROC of 0.75 (*p* = 0.04) in the discrimination between the responders and non-responders groups. The cutoff value of ROC for miR-326 to predict the response of NACT was <0.023, the sensitivity was 88.89%, and the specificity was 50%.

**Conclusions:**

The expression of miR-326 significantly upregulated after NACT in responders. miR-326 may be a biomarker for predicting the response to NACT in LACC patients. The results may optimize individualized treatments for LACC patients.

## Introduction

Cervical cancer ranks second in cancer incidence and death, following breast cancer, in women, with an estimated 570,000 cases and 311,000 deaths in 2018 worldwide (1). The majority of the cases are diagnosed as squamous cell carcinoma (SCC). Human papillomavirus (HPV) infection is the necessary but not sufficient cause of cervical cancer (2). Other important factors are young age at first coitus, multiple sexual partners, high parity, the presence of foreskin in the male partner, cigarette smoke, diet, and so on (3, 4). The risk factors associated with the development of HPV have been summarized in **Table 1**. After Friedlander et al. firstly reported in 1983 that cervical cancer was responsive to cisplatin-based combination chemotherapy (5), neoadjuvant chemotherapy (NACT) followed by radical hysterectomy or radiotherapy has been investigated. NACT can inhibit tumor micrometastases, improve the radiosensitivity of the tumor, and effectively shrink the tumor volume. The ovarian and vaginal functions could be reserved in patients who underwent NACT followed by radical surgery (6). However, the response of NACT varies due to individual differences and the complexity of cancer. Previous studies reported that around 30% of SCC patients were non-responsive to the chemotherapy (7). Identifying responders to NACT could improve their prognosis and optimize individualized treatment. For the non-responders, doctors could stop them from wasting precious time receiving ineffective treatments and suggest other effective treatments.

**Table 1:** Factors linked to the development of cervical cancer.

Behavioral factors	Biological factors
Young age at first coitus (≤16 years old)	Human papillomavirus (HPV)
Multiple sexual partners	Human immunodeficiency virus (HIV)
Long-term oral contraceptive use (≥5 years)	Herpes simplex virus type-2 (HSV-2)
High parity (≥5 times)	Chlamydia trachomatis
Smoking (independent factor)	Neisseria gonorrhoeae
Diet (poor fruit and vegetable intake)	Trichomonas vaginalis
Male characteristics: Presence of foreskin ·HPV DNA positive ·Number of sexual partners ·Experience with sex workers	Treponema pallidum
Immunosuppression	Cytomegalovirus (CMV)

At present, the standard approach for the evaluation of response to NACT is MRI. However, the use of MRI increases the risk of false-positive results and is not a precise way for evaluating the NACT response. Diffusion-weighted MRI can measure the movement of water molecules in tissue, and this molecular imaging can identify the changes during the process of NACT (8). However, it cannot be popularized in developing countries due to the fact that it is costly. Therefore, it is important to identify effective serum biomarkers predicting the NACT response.

MiRNAs are small non-coding RNAs of 18–25 nucleotides that regulate gene expression at the posttranscriptional levels and are involved in the development of multiple malignant tumors (9). Recent studies have discovered that miRNAs may play a key role in predicting response to NACT in cancers (10, 11). The main idea of this study was to find out the potential miRNAs that can predict the response to NACT in LACC patients.

## Methods

### Study design

**Figure 1:**
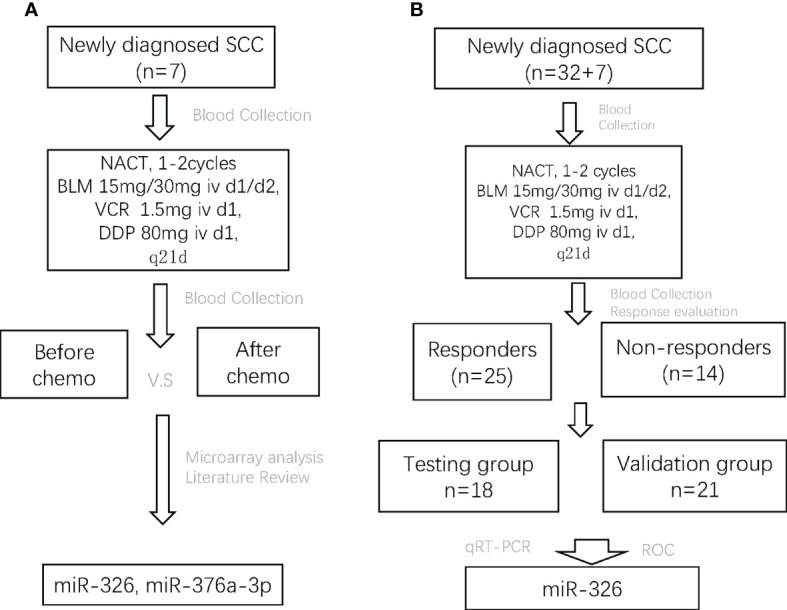
The flowchart of the whole study design. SCC, squamous cell carcinoma; NACT, neoadjuvant chemotherapy; BLM, bleomycin; VCR, vincristine; DDP, cisplatin; CR, complete response; PR, partial response; SD, stable disease; PD, progressive disease.

This study aimed to discover certain circulating miRNAs that could serve as markers for response to NACT in LACC patients. First, the serum samples of seven SCC patients before and after one or two cycles of NACT were collected for microRNA microarray analysis. miRNAs were selected after bioinformatics analysis and literature review. Second, the serum samples of 32 SCC patients before NACT and after one to two cycles of NACT were collected. Third, according to the response evaluation criteria for solid tumors (RECST, version 1.1), SCC patients with complete or partial response were assigned into the chemo-sensitive group (responders) and those with stable or progressive disease into the chemo-insensitive group (non-responders). Fourth, candidate miRNAs were validated both in the responders and non-responders using qRT-PCR analysis. Finally, 18 patients were randomly assigned into the testing group, and the remaining 21 patients were assigned into the validation group. Overall, the study was designed (**Figure 1**).

### Patients and samples

All patients were recruited from the West China Second University Hospital (Chengdu, P.R. of China) between July 2014 and June 2017. This study was approved by the Ethics Committee of West China Second University Hospital. Informed consent was obtained from all participants. All methods were performed in accordance with the relevant guidelines and regulations. All of the participants were genetically unrelated, were ethnic Han Chinese, and donated 5 ml of peripheral blood in every collection. The inclusion criteria were as follows: (1) newly diagnosed with SCC, stages IB–IIB, according to the 2009 FIGO classification; (2) had no prior hysterectomy, pelvic radiotherapy, or systemic chemotherapy; (3) had no other metabolic diseases, such as diabetes mellitus; and (4) had no any inflammations. The basic clinical information of patients is shown in **Table 2**. The SCC diagnosis and staging were assessed by two experienced pathologists. Once the samples were collected, tubes were kept upright at room temperature for 30 min and then stored in a 4°C refrigerator. After centrifugation at 4000 rpm, 4°C for 10 min, the serum was extracted and distributed into aliquots of 1 ml per 1.5 ml tube. Then the serum tubes were stored in a -80°C freezer.

**Table 2:** The clinical characteristics of SCC patients.

	Responders (*n* = 25)	Non-responders (*n* =14)	*p*-value
Age	44.28 ± 9.05	43.64 ± 7.12	0.82
Stage			0.17
Ib	6 (24%)	6 (43%)	
II a	15 (60%)	4 (29%)	
Iib	4 (16%)	4 (29%)	
Differentiation			0.9
Low	22 (88%)	12 (86%)	
Moderate	3 (12%)	2 (14%)	
LNM			0.54
Yes	3 (12%)	0	
No	22 (88%)	14 (100%)	
Stromal invasion			0.45
Yes	5 (20%)	5 (36%)	
No	20 (80%)	9 (64%)	
LVSI			0.72
Yes	7 (28%)	3 (21%)	
No	18 (72%)	11 (79%)	
Parametrial involvement			0.73
Yes	10 (40%)	4 (29%)	
No	15 (60%)	10 (71%)	

### NACT and surgery

Eligible patients received two cycles of NACT in the form of a regimen consisting of bleomycin, vincristine, and cisplatin. Once every 21 days, the patients received bleomycin at 15/30 mg intravenously (iv) on the first day and second day with vincristine at 1.5 mg iv and cisplatin at 80 mg iv on the first day. The doses and schedules of the drug administration were modified according to a drug toxicity evaluation before each course. Radical hysterectomy plus pelvic lymphadenectomy were performed after two cycles of chemotherapy.

### Response evaluation

The response evaluation was based on the response evaluation criteria for solid tumors (RECST, version 1.1) (12) and was classified into four categories—CR: complete response, all the lesions disappeared (any pathological lymph nodes must have a reduction in short axis to <10mm); PR: partial response, at least a 30% decrease in the sum of diameters of lesions; PD: progressive disease, at least a 20% increase in the sum of diameters of lesions; and SD: stable disease, neither a shrinkage that qualified as the PR nor a sufficient increase that qualified as the progressive disease (PD). In our study, responders (chemo-sensitivity) were defined as CR plus PR; non-responders (chemo-insensitivity) were SD plus PD.

### miRNA microarray analysis

RNA was extracted from the serum using a miRNeasy Serum/Plasma Kit (Qiagen, Hilden, Germany) according to the manufacturer’s instructions. RNA was quantified and its purity evaluated by the absorption ratio at 260/280 nm using NanoDrop 2000 (Thermo Fisher Scientific, Massachusetts, USA). The ratio of 260/280 varied from 1.8 to 2.1. Then, cDNA synthesis was performed using a miScript II RT Kit (Qiagen, Hilden, Germany). The expression levels of 192 human mature miRNAs were examined using the miRCURY LNA™ Universal RT microRNA PCR system, Serum/Plasma Focus microRNA PCR Panel (Exiqon, Vedbaek, Denmark). Microarrays were scanned using the ABI PRISM7900 system (Applied Biosystems, Foster, USA), and fold changes in the miRNA expression between the two groups were calculated using the 2-ΔCt method. Raw data were normalized by quantile normalization and the robust multichip average algorithm. All miRNA level files were imported into GeneSpring 11.0 software (Agilent Technologies, Santa Clara, CA, USA).

### Quantitative real-time PCR assay

The relative expression of two miRNAs was validated by qRT-PCR assay. RNA was extracted from the serum using a miRNeasy Serum/Plasma Kit (QIAGEN, Inc.) according to the manufacturer’s instructions. The reverse-transcription reactions were carried out by a miScript II RT Kit (QIAGEN, Inc.). qRT-PCR was performed using a miScript SYBR Green PCR Kit (QIAGEN, Inc.). Glyceraldehyde 3-phosphate dehydrogenase (GAPDH) was used for the internal control of miRNA. Each sample was run in three independent experiments in triplicate. The reactions were amplified at 95°C for 15 min followed by 40 cycles at 94°C for 15 s and 55°C for 30 s.

### Statistical analysis

Quantitative variables were expressed as mean ± SD, and categorical variables were the number of events (%). GraphPad Prism (Version 7, GraphPad Software Inc.) was applied for data analysis with all data assessed for a normal distribution and equal variance. Statistical comparisons between two groups were performed by Student’s *t*-test or the chi-square test. The difference in miRNA levels between groups was evaluated using the Mann–Whitney unpaired test, and, for before/after comparison within one group, the Wilcoxon matched-pairs signed rank test was used. Receiver operating characteristic (ROC) curves were constructed to evaluate the predictive performance of the potential biomarkers. All *P* values were bilateral, with *P* < 0.05 being statistically significant.

## Results

### Demographic and clinical characteristics

Between July 2014 and June 2017, a total of 39 available SCC patients who met the inclusion criteria were enrolled in this study. Twenty-five (64%) of them were evaluated as PR and were assigned into the responders group, whereas the remaining 14 (36%) patients remained stable and were assigned into the non-responders group. Their demographic and clinical characteristics are shown in **Table 2**. Each characteristic was comparable in both groups.

### miR-326 and miR-376a-3p were selected after microarray analysis

**Figure 2:**
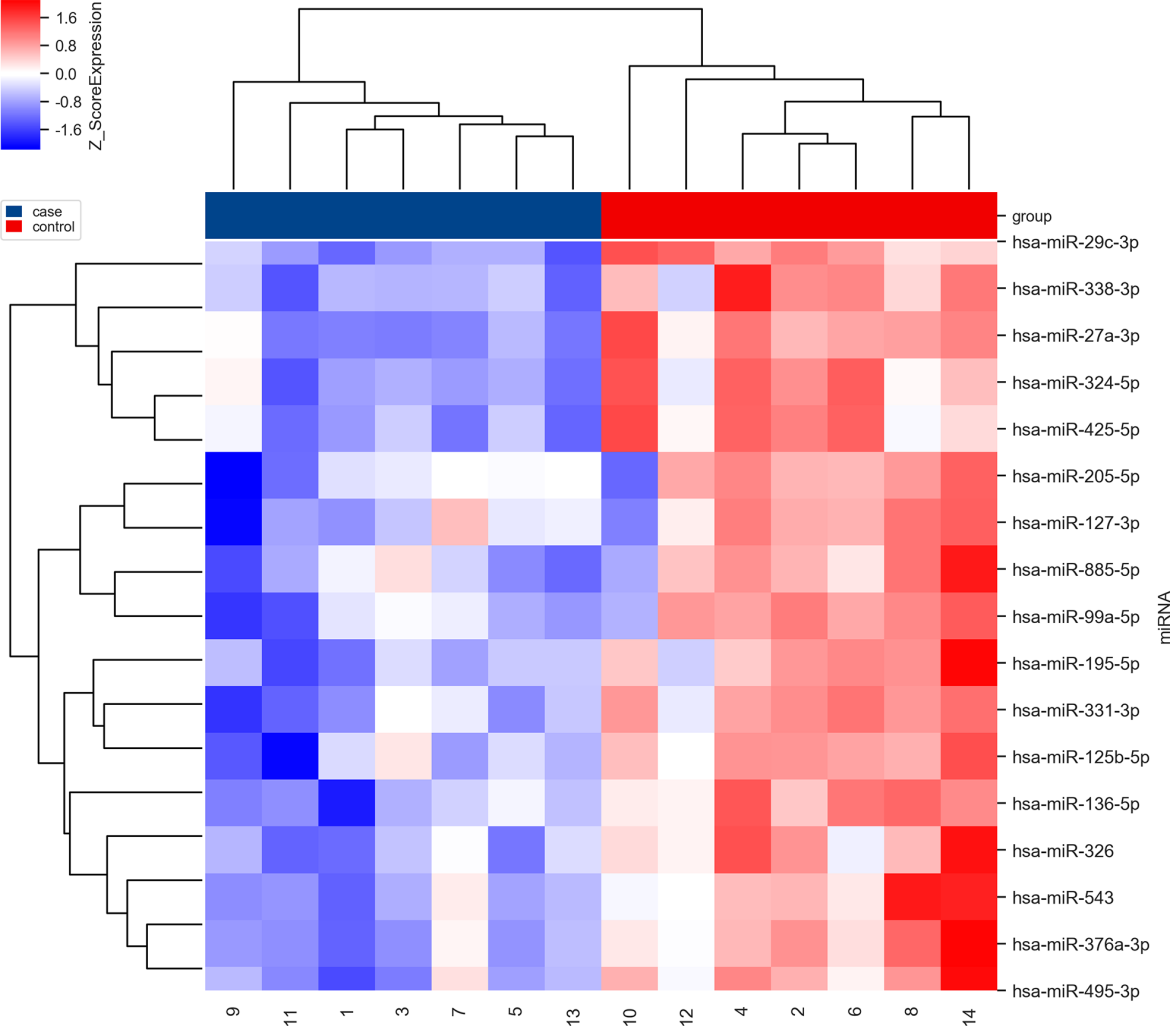
The clustering map of dysregulated microRNAs before (in blue) and after (in red) NACT in the first seven SCC patients. The blue ones mean downregulated and the red ones mean upregulated.

Pair-matched blood samples of seven SCC patients before and after receiving NACT were collected to perform microRNA microarray analysis. Seventeen significantly differentially expressed microRNAs were selected according to bioinformatics analysis in our previous study. These 17 microRNAs were significantly downregulated before NACT and upregulated after NACT (**Figure 2**). Combined with the literature review (13, 14), miR-326 and miR-376a-3p were selected for further explorations.

### Compared with the baseline, the relative expression of miR-326 after NACT was higher and reached the statistical difference in the responders group

**Figure 3:**
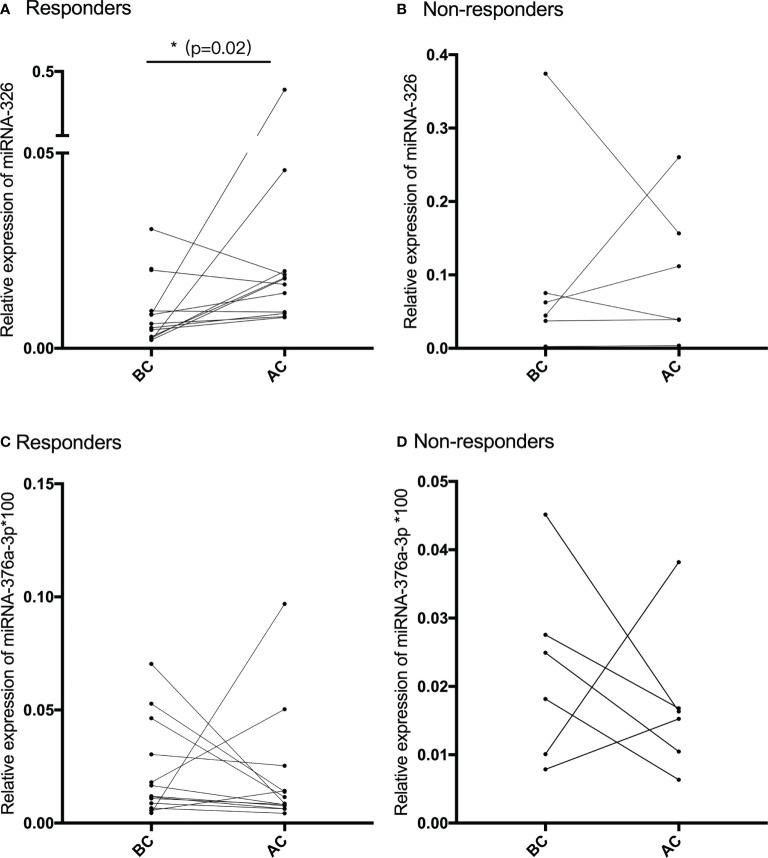
**(A, B)** The relative expression of miR-326 before and after NACT in chemotherapy-sensitive patients (responders) and chemotherapy-resistant patients (non-responders). **(C, D)** The relative expression of miR-376a-3p before and after NACT in chemotherapy-sensitive patients (responders) and chemotherapy-resistant patients (non-responders).

The pair-matched blood samples of 18 SCC patients before and after receiving NACT were used to explore the expression change of miR-326 by using qRT-PCR. According to the new response evaluation criteria in solid tumors (RECST, version 1.1), 12 patients achieved PR and were classified into the responders group, and the remaining six patients achieved SD and were classified into the non-responders group. After the qRT-PCR analysis, miR-326 was significantly downregulated before NACT and upregulated after NACT in 12 responders (*p* = 0.02, **Figure 3A**), whereas the expression before and after NACT had no statistical difference in the non-responders (**Figure 3B**). The expressions of miR-376a-3p before and after NACT had no statistical difference neither in the responders group nor the non-responders group (**Figures 3C, D**).

### miR-326 could be a biomarker to predict response to NACT in SCC patients

**Figure 4:**
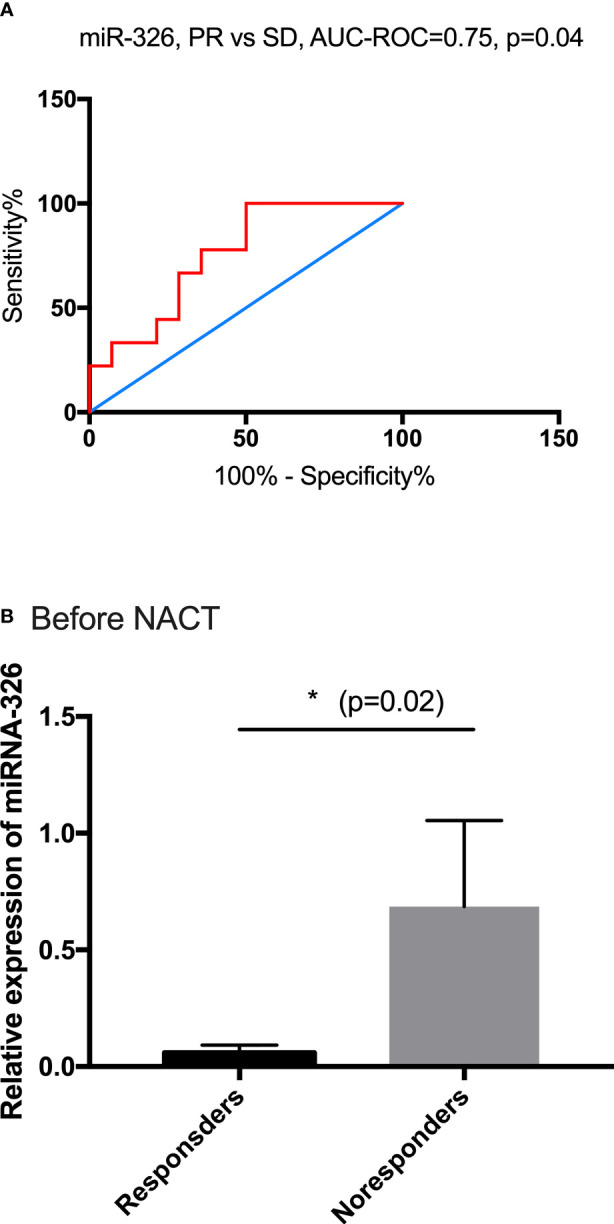
The predictive performance of miR-326. **(A)** The ROC curve for PR and SD based on the relative expression of miR-326, AUC-ROC = 0.75. **(B)** Before NACT, the baseline expressions of miR-326 in responders were much lower than those in non-responders (*p* = 0.02).

miR-326 was selected to be the candidate biomarker for predicting the NACT response and was validated by 21 SCC patients in a further analysis. Among them, 13 patients achieved PR, and the remaining eight patients were in the SD group. The predictive performance was evaluated by sensitivity, specificity, accuracy, and the area under the ROC. miR-326 provided an AUC-ROC of 0.75 (*p* = 0.04) in the discrimination between the PR and SD groups (**Figure 4A**). The cutoff value of ROC for miR-326 to predict the response of NACT was <0.023, the sensitivity was 88.89%, and the specificity was 50%. Before NACT, the relative expression of miR-326 in the responders was much lower than that in the non-responders (*p* = 0.02) (**Figure 4B**).

## Discussion

In a meta-analysis, NACT followed by radical surgery showed a highly significant reduction in the risk of death compared with radiotherapy alone (hazard ratio [HR] = 0.65, 95% CI [0.53, 0.8], *P* = 0.0004), with an absolute improvement of 14% in survival at 5 years, increasing from 50% to 64% (15). However, many patients cannot receive follow-up surgery because of drug toxicity or insensitivity to NACT resulting to delaying the time to receive chemoradiotherapy. Therefore, it is meaningful to find some predictive biomarkers to predict the response to NACT in newly diagnosed LACC patients.

MicroRNAs are stable and detectable in the serum, and changes in the levels of specific microRNAs could allow the detection of clinical conditions (16, 17). Feng et al. reported that miR-15a, miR-16-1, miR-29c, miR-34a, and miR-155 could be novel non-invasive biomarkers for the diagnosis of diffuse large B-cell lymphoma (DLBCL) with AUC-ROCs of 0.7722, 0.7002, 0.6672, 0.8538, and 0.7157, respectively (18).

Searching for effective prevention and management of cancers has always been at the top of medical concerns list. MicroRNAs (miRNAs), less than 22 nt small non-coding RNAs, play important roles by regulating mRNAs in cancer-related processes (9). There are still some small non-coding RNAs linked to other gynecologic cancers, which have been shown to contribute positively to the prognosis prediction of oncology patients. Piergentili et al. summarized ncRNAs with good predictive values such as miR-101, miR-152, and miR-205 in the treatment of endometrial cancer and recognized their great potential to improve risk stratification in EC (19). Cavaliere et al. also identified ncRNA pools that have a prognostic role and impact on the treatment of EC patients based on epigenetic profiles (20). However, the molecular properties of individuals may not be generalized due to the interactions between different ncRNAs. Focusing on only one molecule may not be sufficient, and combining ncRNAs with other well-known risk factors may be the key to achieving better treatment approaches. Researchers demonstrated that in luminal B HER-2 breast cancer, the miR-718 and miR-4516 expressions were lower in the group of responders than in non-responders, whereas the miR-210 expression was the opposite (21). Not come singly but in pairs, miR-195 and miR-26b were also found to be consistently upregulated after NACT (22). Todorova and colleagues found that miR-328-3p expression was downregulated before NACT and suggested that miR-127-3p is a strong predictor of NACT-positive treatment response in triple-negative breast cancer (23). Furthermore, miRNA-21 could be used as an independent predictor of chemotherapy sensitivity not only in breast cancer but also in esophageal SCC, where its levels were remarkably lower in chemotherapy-sensitive patients, whereas levels in the non-responders did not change significantly (24, 25). This indicates that miRNAs that are predictable for chemotherapy response may not only target single cancer.

Plenty of studies have proved that dysregulations of miR-326 are associated with pathological processes such as cellular proliferation and apoptosis (26), differentiation, metastasis (27, 28), and chemotherapy resistance (29, 30). Our study found that circulating miR-326 in LACC patients who were sensitive to chemotherapy significantly downregulated after NACT, whereas the expression in chemotherapy-resistant patients had no statistical difference between before NACT and after NACT. The results implied that circulating miR-326 in newly LACC patients could be a biomarker to predict the response to NACT. Liang et al. showed that miR-326 was downregulated in VP-16-resistant multidrug resistance cell lines and in a panel of advanced breast cancer tissues and consistent reversely with the expression levels of MRP-1. These results demonstrated that miR-326 could be an agent in the mechanism of chemotherapy resistance of breast cancer (29).

There are several studies that focus on exploring biomarkers or others to predict the response to NACT of newly diagnosed cervical cancer patients. Yan Hou et al. identified and verified L-valine and L-tryptophan as the biomarkers to predict the response to NACT by performing plasma metabolite profiling (31). Chun Fu et al. studied in a different angle and found that the axial and sagittal magnetic resonance diffusion-weighted imaging (DWI) could detect the changes in LACC patients after NACT, and the apparent diffusion coefficient values measured could be used to evaluate the response to NACT (8).

The strengths of this study are based on the meta-analysis; the target miRNAs are selected by microarray analysis, and then we screen differentially expressed miRNAs in the serum of SCC patients, which are more stable and have less influence during the collection of samples.

The limitations of our work are that we just found the potential biomarker, and we failed to find the correlation between miRNAs expression and HPV infection, as HPV infection is widely regarded as the primary cause of SCC.

Cervical adenocarcinoma patients should be included in the following relevant studies, which will benefit further clinical application. Moreover, the target gene, protein, and regulating pathway need to be explored. Finally, a large population is also needed in future research to validate the predicted performance of miR-326 in SCC patients.

## Conclusions

In summary, our present study provides the first evidence that the circulating miR-326 is significantly upregulated after receiving NACT in responders, whereas the expression has no change in non-responders. The cutoff value of ROC for miR-326 to predict the response of NACT was <0.023. It suggested that the expression of miR-326 was lower than 0.023 in patients with newly diagnosed cervical SCC, indicating that they were likely sensitive to the NACT. It seems that the circulating miR-326 may be a biomarker to predict the response of NACT in SCC patients.

## Data availability statement

The raw data supporting the conclusions of this article will be made available by the authors, without undue reservation.

## Ethics statement

The studies involving human participants were reviewed and approved by the Ethics Committee of West China Second University Hospital. The patients/participants provided their written informed consent to participate in this study.

## Author contributions

KZ contributed to the design of the study, sample collection and preparation, interpretation of data, manuscript drafting, and final approval. EY contributed to the sample collection and preparation and bioinformatics analysis. TC contributed to sample collection and preparation. ZL contributed to the conception of the study and design, manuscript revision, and critical discussion. All authors contributed to the article and approved the submitted version.

## Funding

The microarray work and analysis of the data of this study were supported by the Medical Science and Technology Project of Sichuan Province Health Commission (No.21PJ050).

## Acknowledgments

We acknowledge the staff of the Key Laboratory of Birth Defects and Related Diseases of Women and Children (Sichuan University), Ministry of Education, Chengdu, People’s Republic of China, for the methods instruction of the study.

## Conflict of interest

The authors declare that the research was conducted in the absence of any commercial or financial relationships that could be construed as a potential conflict of interest.

## Publisher’s note

All claims expressed in this article are solely those of the authors and do not necessarily represent those of their affiliated organizations, or those of the publisher, the editors and the reviewers. Any product that may be evaluated in this article, or claim that may be made by its manufacturer, is not guaranteed or endorsed by the publisher.

## Abbreviations

LACC, locally advanced cervical cancer; NACT, neoadjuvant chemotherapy; miRNAs, microRNAs; ROC, receiver operating characteristic; qRT-PCR, quantitative reverse transcription–polymerase chain reaction; SCC, squamous cell carcinoma; HPV, human papilloma virus; CR, complete response; PR, partial response; PD, progressive disease; SD, stable disease; DLBCL, diffuse large B-cell lymphoma.
